# Comparison of two GP service provider models in older adults: a register-based follow-up study

**DOI:** 10.3399/BJGPO.2022.0101

**Published:** 2023-06-14

**Authors:** Aina Enckell, Merja K Laine, Hannu Kautiainen, Mika T Lehto, Kaisu H Pitkälä, Ossi Rahkonen, Hanna-Maria Roitto, Timo Kauppila

**Affiliations:** 1 Department of General Practice and Primary Health Care, University of Helsinki and Helsinki University Hospital, Helsinki, Finland; 2 City of Espoo, Welfare and Health Sector, Espoo, Finland; 3 Folkhälsan Research Centre, Helsinki, Finland; 4 Primary Health Care Unit, Kuopio University Hospital, Kuopio, Finland; 5 City of Vantaa, Vantaa, Finland; 6 Department of Public Health, University of Helsinki, Helsinki, Finland; 7 Department of Neurosciences, University of Helsinki, Helsinki, Finland; 8 Department of Geriatrics, Helsinki University Hospital, Helsinki, Finland; 9 Population Health Unit, Finnish Institute for Health and Welfare, Helsinki, Finland

**Keywords:** continuity of patient care, follow-up, general practitioners, health services accessibility, patient appointments, primary health care

## Abstract

**Background:**

In Finland, there have been various strategies attempting to provide access to GPs. The 'restricted-List General Practitioner model' (rLGP) was launched in primary health care (PHC) in the city of Vantaa after the 'named General Practitioner model' (nGP) failed to provide sufficient access to GPs. This was done to improve access to GP appointments for those most needing care.

**Aim:**

To evaluate the impact of the transition from nGP to rLGP on access to non-urgent scheduled appointments among patients aged ≥75 years.

**Design & setting:**

A register-based follow-up study in public PHC in Vantaa, Finland.

**Method:**

The study focused on patients aged ≥75 years who used PHC from 2004–2008. It looked at the number of non-urgent and urgent scheduled appointments, patient contacts, home visits, PHC emergency department appointments, and cancelled appointments, which were recorded 7 years before and after the transition from nGP to rLGP in 2011 and adjusted to patient-years. Non-urgent appointments were booked to the patient’s own nGP or rLGP in public PHC, whereas urgent appointments could be to any GP.

**Results:**

The number of non-urgent scheduled appointments to GPs was halved during the time of nGP, before launching the rLGP. Simultaneously, the number of urgent scheduled appointments more than tripled. The number of both started to plateau a year before the rLGP was launched. The number of both non-urgent and urgent scheduled appointments remained mainly at that level after rLGP was implemented.

**Conclusion:**

The rLGP model was unsuccessful in improving access to non-urgent scheduled appointments to GPs.

## How this fits in

A well-functioning service provider model could provide a good basis for adequate access and continuity of care in PHC. Two models were compared that were used in public PHC centres in the city of Vantaa, Finland. It was found that the transition from a named GP model to a restricted-list model did not improve access to non-urgent appointments to the GP.

## Introduction

Adequate access and continuity of care are considered essential for good quality PHC.^1,2^ Access to PHC is important when attempting to improve the health and wellbeing of the population, and to further reduce mortality rates.^3–5^

In Finland, there have been various strategies in PHC that have attempted to improve access to GPs. PHC services are mainly organised by municipal PHC centres that are publicly funded, and the GPs are, for the most part, employed and directly governed by the municipal health administration.^6^ The size of PHC centres and the population assigned to them vary considerably.

Employed patients may choose to consult a GP either in PHC or in occupational health,^7^ as occupational health services — expenses largely covered by employing companies — also play a notable role in Finnish health care by providing primary care to the employed population. Occupational health services end when the employee retires, which in Finland is at an age of approximately 65 years. Thus, PHC is the main provider of health care for older adults if they do not choose a private physician, for which they must pay extra.^8^

In the 1990s, the city of Vantaa organised PHC based on the nGP (a named GP model).^9^ The idea of the nGP was that the GP had a geographic area with a population of around 2000 people whose health they were responsible for.^9^ GPs took care of all primary care health problems of their population, including, to some extent, preventive medicine. GPs during this time experienced high workloads and professional demands, and poor possibilities for colleague consultation.^10,11^ Based on a Finnish study, patients initially liked the nGP system because of the continuity of care,^12^ but the access to care gradually worsened over the years, as the named GPs in Vantaa started to transfer from PHC centres to other workplaces owing to high workload.^13–16^ As the GP workforce decreased and the total number of appointments was reduced, patients had to substitute their non-urgent scheduled appointments with their nGP for urgent scheduled appointments with any GP, which led to poor continuity of care. Furthermore, it became challenging to employ GPs to work in the PHC centres^15^ and, finally, the economic recession of 2008 reduced financial resources^17^ in the municipalities. Therefore, in 2011, the municipality of Vantaa launched another model — the rLGP (a restricted-list GP model) — to ensure access to GPs and continuity of care for older adults aged ≥75 years and those suffering from chronic diseases.^15,18^ This rLGP was adapted from PHC in neighbouring countries, where patients were able to enlist to a GP if there were open positions.^19,20^ The Norwegian enlistment system was reported to be associated with good patient and GP satisfaction, adequate access to GPs, relatively well-functioning communication between patients and GPs, and good continuity of care in PHC.^19,21–24^ Furthermore, this kind of enlistment was reported to be associated with decreased use of emergency services.^25^ Thus, the theory was that the new rLGP model, adapted from neighbouring countries, would ensure access to care for the patients who needed it the most, by attracting a GP workforce and by allocating non-urgent appointments for the listed patients.^18^

To be eligible for enlistment to the rLGP in Vantaa, the patient had to meet one of the following criteria: to be aged ≥75 years; to have had ≥7 appointments to a GP during the previous 2 calendar years; or have a certain chronic diagnosis that requires constant management (for example, diabetes or heart disease). Additionally, a patient could also be enlisted if a healthcare professional judged that they would benefit from it. Patients, other than the above mentioned, were not assigned to any particular GP and were instead taken care of by the GP teams with collective responsibility.

In the city of Vantaa, the transition from the original nGP to the novel rLGP occurred in 2011. The aim of the study was to evaluate the impact of the transition from nGP to rLGP on access to non-urgent scheduled appointments among patients aged ≥75 years.

## Method

This register-based follow-up study was conducted in the city of Vantaa, Finland. With a population of 239 206 (as of 31 December 2021), Vantaa is the fourth most populated city in Finland, located in the Helsinki metropolitan area. Of the whole population, 50.2% were men, 15.7% were aged ≥65 years, and 23.4% had a foreign background. Of the older population (≥75 years), 40% were men.^26^

Data for the Vantaa PHC centres were obtained from the Graphic Finstar electronic health record system. This electronic health record system enabled the data to be analysed at the individual patient and GP levels. Data consisted of all face-to-face appointments and patient contacts during the study period between 1 September 2004 and 31 August 2018. Consultations were used as a proxy measure of access. Data were collected from all of the seven public PHC centres in Vantaa. Data from the private sector were not available.

The main measures were the number of various types of GP consultations documented 7 years before and after the transition from nGP to rLGP. The transition from nGP to rLGP took place on 1 September 2011. The following were calculated: the number of face-to-face scheduled non-urgent appointments and urgent appointments; paper and phone consultations; home visits; PHC emergency department appointments; and cancelled appointments.

A non-urgent appointment with sufficient time was a consultation performed by the named or list GP. This appointment was long enough to enable comprehensive care of chronic diseases. An urgent appointment was a short consultation by any GP in the health centre. A paper consultation was a note the GP writes in the electronic health record system without seeing the patient or talking to them on the phone; for example, interpretation of laboratory results containing care instructions, or statements.

The appointments were booked preferably to the named or list GP by phone or at the PHC centre by a healthcare worker, and were the same at all seven PHC centres during the study period. If there were any technical problems with the electronic health record system, the GPs wrote and added the appointment records afterwards. The number of various types of consultations were adjusted to the number of individuals aged ≥75 years in the population using the service. As a secondary measurement, the amount of GP workforce, expressed as person-years, was adjusted to the number of the same population.

### Statistical analyses

Incidence rates of consultations were calculated across 14 calendar-year periods (2004−2018), 7 years before and after intervention. Consultations of GPs were reported as appointment rates per 1000 person-years with 95% confidence intervals (CIs) based on a Poisson distribution. The Poisson regression models were tested using the goodness-of-fit test, and the assumptions of overdispersion in models were tested using the Lagrange multiplier test. A possible non-linear relationship between the number of contacts and the years was assessed by using a 4-knot-restricted cubic spline Poisson regression model. Stata (version 17.0) statistical package was used for the analysis.

## Results

Altogether, 1 392 266 GP consultations from 23 409 different patients were recorded. Of the patients, 39.1% were men. The mean age at the time of appointments was 81 years (standard deviation: 5 years). Data consisted of 211 184 non-urgent and 149 978 urgent scheduled appointments to GPs’ offices, 9724 home visits performed by the GPs, 531 750 paper consultations, 156 149 phone consultations, 281 950 prescription renewals, 49 424 visits to the PHC emergency department, and 2107 cancelled appointments to the GPs.

**Figure 1.**
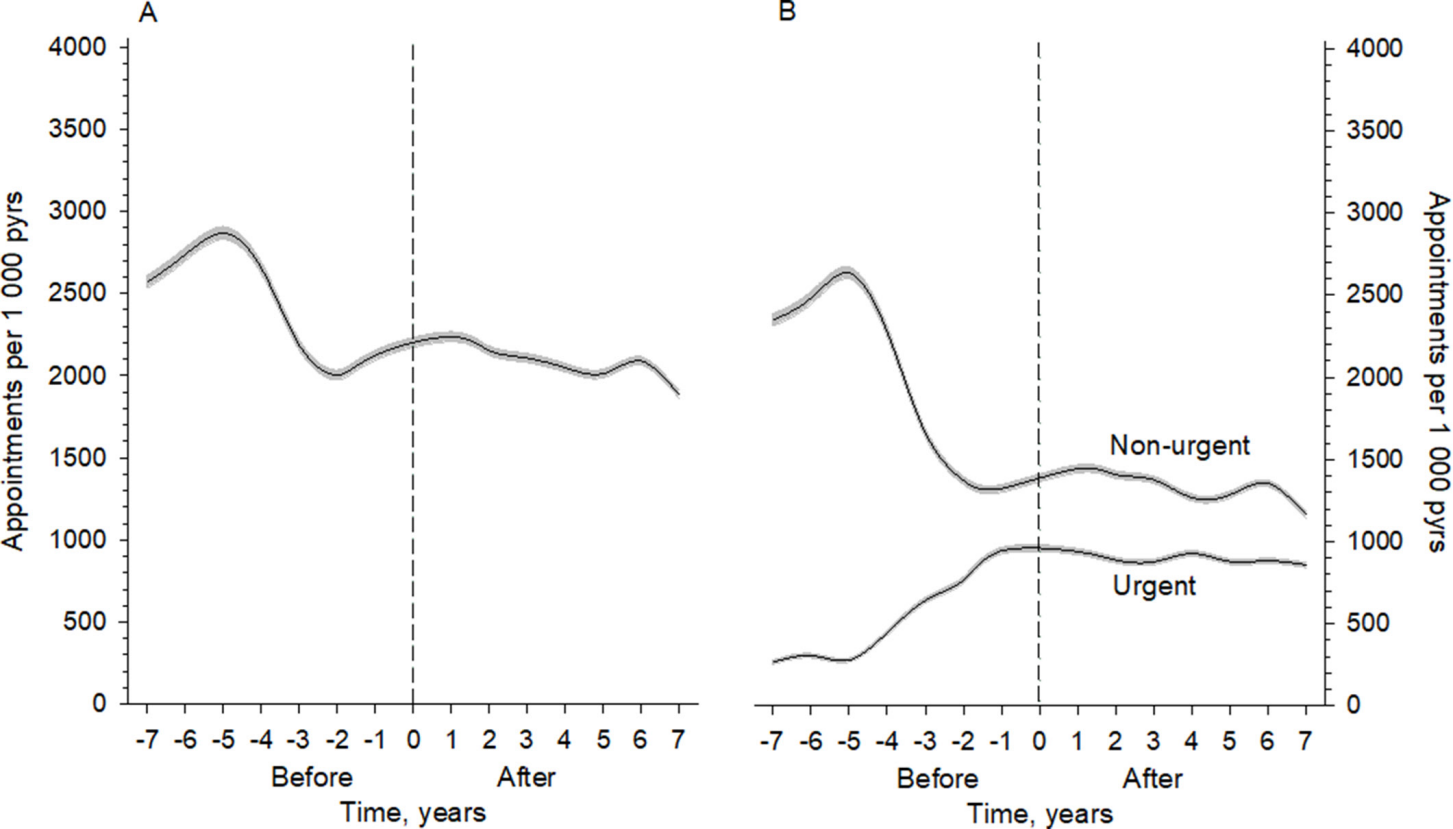
**(A**) Numbers of all face-to-face appointments of patients aged ≥75 years to GPs per 1000 person-years. The line shows the mean, and the dashed area the 95% confidence interval. (**B**) Numbers of non-urgent scheduled and urgent scheduled appointments to GPs per 1000 person-years. The appointments were derived from 4-knot-restricted cubic spline regression models. Grey areas represent 95% confidence intervals. pyrs = person-years; 0 = timepoint of model change.

The number of all appointments decreased during the nGP (*P*<0.001); they had, however, already plateaued before the transition to rLGP (Figure 1a). The number of non-urgent scheduled appointments to the GP plateaued following the same pattern (Figure 1b) (*P*<0.001). The increase in the number of urgent scheduled consultations occurred before rLGP was implemented (Figure 1b).

**Figure 2.**
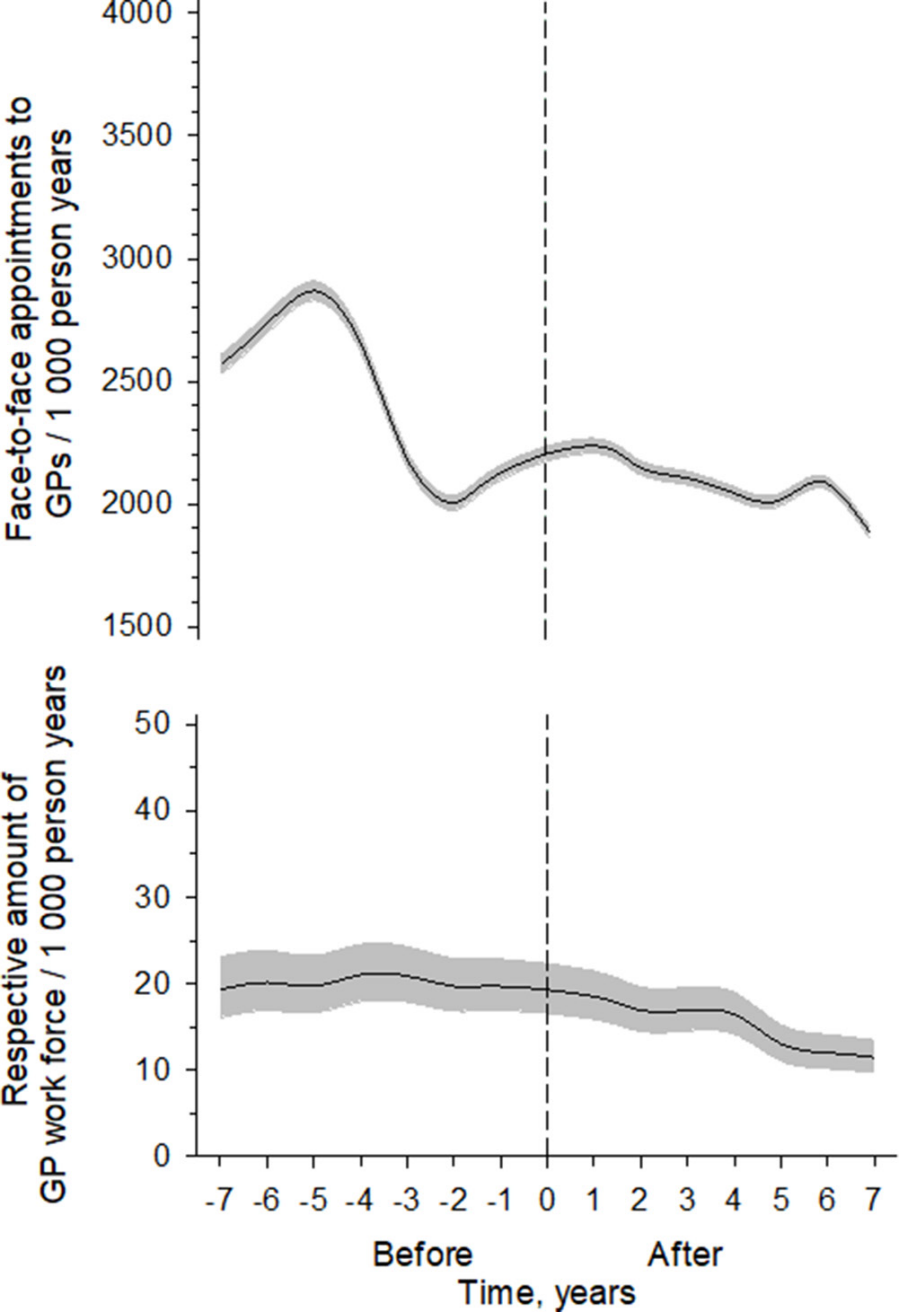
The top figure illustrates the numbers of all face-to-face appointments to GPs per 1000 person-years and the bottom figure the respective amount of GP workforce expressed as person-years adjusted to the number of patients aged ≥75 years. The appointments were derived from 4-knot-restricted cubic spline regression models. Grey areas represent 95% confidence intervals. pyrs = person-years; 0 = timepoint of model change.

Implementing rLGP had no impact on home visits performed by the GPs (data not shown) or appointments at the PHC emergency department (data not shown). Implementing rLGP did not alter the number of paper or phone consultations or cancelled appointments to the GPs (data not shown). The work resources of GPs adjusted to the studied population decreased during the rLGP period (Figure 2) (*P*<0.001).

## Discussion

### Summary

The transition from nGP to rLGP did not significantly alter access to PHC services in patients aged ≥75 years. The workforce of GPs adjusted to the studied population decreased, especially at the end of the follow-up time. Although the study population had a named GP in both models, the transition to rLGP failed to improve access to non-urgent appointments.

### Strengths and limitations

The study has various strengths. The present data were very large and precise, and provided lots of information about the quantitative changes from the administrative point of view. Of the patients, 39.1% were men, so in terms of sex, the studied population represented the older adults in Vantaa well. As women tend to live longer, they are usually over-represented in studies of this age group.^26^ The data allowed the authors to study various types of appointments to the GPs. The follow-up period was relatively long; therefore, it provided the possibility to make reliable conclusions about the trends in the studied parameters. Although similar models have been applied in various cities and municipalities in Finland, comparative research between the models has not been done before. It is important to assess the effects of any changes in PHC and evaluate the consequences.

As a limitation, the present study does not provide information about the qualitative aspects of these models from the healthcare professionals’ or patients’ perspectives. Data on whether urgent appointments were appropriate would have helped to determine whether access to non-urgent appointments was inadequate and, therefore, led to an increase in inappropriate acute appointments being booked. A consultation is an administrative unit that allows the resources used to be counted, but it does not provide information about the output of the studied PHC, such as endpoint-level information about the impact on health, diseases treated, or healthcare professionals’ permanence.^27,28^ The time of the appointment captured the amount of time that was booked, not the actual time used. Thus, it is not known if the time spent with the patient altered pre- and post-intervention. Further, it is not known how often the patients saw the same GPs during the non-urgent or urgent appointments. Lastly, the study had no reliable data on nurse appointments before the year 2008. This information would have been necessary to evaluate whether there was a shift of workload from GPs to nurses.

### Comparison with existing literature

In the beginning of the follow-up in 2004–2006 when the nGP was still functioning well, the level of non-urgent scheduled appointments was relatively high and urgent appointments low. After those years, it became challenging to respond to the population’s health needs, and there was a shift from non-urgent appointments towards urgent appointments. It has been suggested that if PHC is unavailable, more urgent treatment-oriented sites of health care are forced to back-up its functions.^29,30^ In line with this, when PHC started to have difficulties maintaining its normal non-urgent services during the last years of nGP,^14,17^ non-urgent scheduled appointments were compensated for by urgent ones, although appointments at the emergency department per se were not affected in the study population.

Changing the nGP to rLGP did not increase the number of non-urgent appointments to GPs in the studied population aged ≥75 years, as had been expected based on the positive experiences from rLGP-like models elsewhere.^19,21–32,31,32^ It must, however, be pointed out that although the Norwegian list system acted as an example for the rLGP, there were also differences: in Norway the size of a list was 900–1200 patients, and there were only a few unlisted patients to take care of.^22^ In the present study, the size of a list was 400–500 patients who fulfilled the previously mentioned criteria and, in addition to this, the GPs had appointments with unlisted patients and urgent appointments with all patients, as well as preventive medicine. Therefore, the results are not directly comparable with those yielded in Norway.^19,21–25^ Older patients tend to use PHC because they have more chronic diseases and require more medical attention than younger patients.^33^ According to previous studies, short urgent appointments are not likely to be enough for older adults with various medical concerns.^34–36^ Thus, non-urgent scheduled appointments are more likely to ensure good medical praxis in this patient group.

The poor availability of GPs may be one of the reasons for the decrease in the input to care in the past 4 years of the follow-up, as resources per patient-years decreased in the studied age cohort. Thus, organisational changes did not overcome the lack of resources in PHC. The decrease in workforce did not lead to increased cancellations of non-urgent GP appointments. Further, previous Finnish studies have reported that the number of appointments per GP has generally decreased in PHC.^14,37^ During the past decades, this decrease has not been evident in the oldest-age cohorts.^17^ There was no increase in the other studied types of consultations either. Phone consultations have been suggested to replace face-to-face appointments to GPs,^38^ but no such increasing trend was seen in their use in the study cohort during these years. Although GP home visits might be ideal for treating older patients,^39^ whose treatment was expected to be improved with rLGP, no increasing trend was seen in these services either. The amount of home visits has traditionally been small in Finland because they are difficult and expensive to arrange. Additionally, this study did not observe that older patients were excessively redirected to the PHC emergency department, although this resource has often been suggested as a backup for office-hour primary care.^40^

### Implications for practice

In older adults, rLGP was unsuccessful in the attempt to improve access to non-urgent scheduled appointment to GPs, which are thought to create the basis for comprehensive care.

## Data availability

Data are not available. The ethical board did not allow giving data outside the researchers because they contain personal information about patients and physicians.

## Data

Data are not available. The ethical board did not allow the sharing of data outside the research group because they contain personal information about patients and physicians.
